# An efficient and comprehensive plant glycerolipids analysis approach based on high‐performance liquid chromatography–quadrupole time‐of‐flight mass spectrometer

**DOI:** 10.1002/pld3.183

**Published:** 2019-11-15

**Authors:** Shaoping Lu, Hongbo Liu, Cheng Jin, Qing Li, Liang Guo

**Affiliations:** ^1^ National Key Laboratory of Crop Genetic Improvement Huazhong Agricultural University Wuhan China

**Keywords:** glycerolipid, lipidomics, lipids, mass spectrometry, rapeseed, UPLC‐TripleTOF

## Abstract

In past two decades, numerous lipidomics approaches based on mass spectrometry with or without liquid chromatography separation have been established for identification and quantification of lipids in plants. In this study, we developed an efficient and comprehensive lipidomics approach based on UPLC with an Acquity UPLC^TM^ BEH C18 column coupled to TripleTOF using ESI in positive ion mode and MS/MS^ALL^ scan for data collection. Lipid extract was prepared to 2 mg/ml solution according to dry tissue weight and mixed with 13 kinds of internal standards including PA, PC, PE, and PG. Each analysis required single injection of 5–10 μl lipid solvent and completed in 32 min. A target method dataset was generated using the LipidView software for prediction of the accurate mass of target lipid species. The dataset was uploaded into the PeakView to create processing datasets to search target lipid species, which achieved batch data processing of multiple samples for lipid species‐specific identification and quantification. As proof of concept, we profiled the lipids of different tissues of rapeseed. Thirteen lipid classes including 218 glycerolipids were identified including 46 TAGs, 15 DAGs, 20 PCs, 24 PEs, 13 PGs, 14 PIs, 26 PSs, 12 PAs, 16 MGDGs, 16 DGDGs, 6 LysoPCs, 5 LysoPEs, and 5 LysoPGs. Together, our approach permits the analysis of glycerolipids in plant tissues with simplicity in sample analysis and data processing using UPLC‐TripleTOF.

## 1 INTRODUCTION

Lipids are essential components of cell membranes. Changes in lipids lead to the alterations of the fluidity (Dawaliby et al., 2016; Nguyen, Rudge, Zhang, & Wakelam, 2017), permeability (Colombini, 2017; Van der Paal, Neyts, Verlackt, & Bogaerts, 2016), and polarity (Hammond & Hong, 2018; Tejos et al., 2014) of the cell membrane. In plants, these alterations trigger cell response to environmental stimuli such as drought, salt, cold, nutrition, and pathogen (Hong et al., 2016; Lu et al., 2016; Peppino Margutti, Reyna, Meringer, Racagni, & Villasuso, 2017; Ruelland et al., 2015; Singh, Bhatnagar, Pandey, & Pandey, 2015). In plants, glycerolipids consist of monogalactosyldiacylglycerol (MGDG), digalactosyldiacylglycerol (DGDG), triacylglycerol (TAG), diacylglycerol (DAG), and different types of phospholipids (Wei, Fanella, Guo, & Wang, 2016; Zheng et al., 2017). Phospholipids are essential components of biological membranes and participate in plant signal transduction process (Nakamura, 2017). Triacylglycerol supplies energy and essential fatty acids for human health (Calder, 2015; Song et al., 2018; Spector & Kim, 2015), and it also supplies energy for plant seed germination and maintains cellular energy balance for plant growth (Tsai et al., 2015; Xu & Shanklin, 2016; Yang & Benning, 2018). MGDG and DGDG are the major components of the membrane system in chloroplast (Rocha et al., 2018), while DAG plays key roles in cell signaling as a second messenger and a precursor of TAG (Vermeer et al., 2017). Thus, quantitative determination of glycerolipids is becoming important for understanding lipids metabolism and their functions in plants (Peng & Ahrends, 2016). However, quantifying and identifying lipid species accurately and comprehensively is a challenging task due to their complex structure and variation in content (Caforio & Driessen, 2017; Ganzera & Sturm, 2018; Jouhet et al., 2017; Moellering, Prince, & Prince, 2017; Zheng et al., 2017).

In past two decades, lipidomics approaches have been established with the development of mass spectrometry platforms (Ganzera & Sturm, 2018). Electrospray ionization tandem mass spectrometry (ESI‐MS/MS) represents the most popular and classic technique in the area of lipidomics analysis which is well known for its sensitivity and accuracy (Devaiah et al., 2006; Tarazona, Feussner, & Feussner, 2015; Welti et al., 2002). There are two alternative modes, direct infusion and liquid chromatography (LC), to introduce sample in the ESI source (Gao et al., 2016, 2018; Simons et al., 2012). A direct infusion approach (also called shotgun lipidomics) based on electrospray ionization mass spectrometry (ESI‐MS/MS) using precursor, and neutral loss scan mode was developed by Welti et al. (2002) which has been widely used in plant lipids analysis (Devaiah et al., 2006; Han, Yang, Yang, Cheng, & Gross, 2006; Welti et al., 2002; Welti, Wang, & Williams, 2003). This approach was successfully used to analyze phospholipids and galactolipids (Devaiah et al., 2006; Ejsing et al., 2009; Li, Welti, & Wang, 2006; Peters et al., 2010). However, DAG and TAG analyses required additional analytical approaches (Li, Baughman, et al., 2014; Li, Butka, & Wang, 2014). Triple quadrupole‐based ESI‐MS/MS approach with or without LC separation using multiple reaction monitoring (MRM) mode is another proven method to comprehensively analyze targeted lipids (Brugger, 2014; Cheong, Wenk, & Shui, 2014; Junza, Amatya, Barron, & Barbosa, 2011; Vu et al., 2014). Conventional ‐based ESI‐MS/MS approaches require preselection characteristic ions and are highly targeted (Gao et al., 2016, 2018; Simons et al., 2012). In addition, they employed deconvolution in their quantification schemes, because the detected intensities include signal contributed by isotopomers, and, in some cases, doubly charged lipids and their isotopomers (Gao et al., 2018; Groessl et al., 2014; Han et al., 2006; Welti et al., 2002, 2003).

Recently, high‐resolution mass spectrometers such as Obitrap and time of flight (TOF) were used for lipid analysis because of mass accuracy, fast spectral acquisition rate, high resolution, and high sensitivity (Andrews, Simons, Young, Hawkridge, & Muddiman, 2011; Stahlman et al., 2009). However, the quantification is relative and many lipid species were missing in their analysis (Li et al., 2017; Zheng et al., 2017; Zhou et al., 2017). A developed analysis technique using a hybrid quadrupole high‐resolution time of flight (TripleTOF) and data collection with MS/MS^ALL^ scan based on data‐independent acquisition greatly improves the coverage of lipids (Gao et al., 2017, 2016, 2018). TripleTOF is built for fast scanning, high resolution, and accuracy, and its sensitivity is comparable to mass spectrometer (Gao et al., 2016; Zheng et al., 2017). Gao et al. (2017) identified more than 1,200 lipid species in a dried blood spot using a shotgun lipidomics method, and the species of phosphatidylglycerol and monoacylglycerol can be quantified less than 6 min (Gao et al., 2017, 2018). In plants, 144 glycerolipid species of yellow sarson seeds were relatively quantified in 22 min performed on UPLC‐TripleTOF platform with ESI in positive ion mode (Zheng et al., 2017). After data collection by high‐resolution mass spectrometers, lipid analysis is usually carried out by searching against database which typically generates false‐positive results which require expertise for manual correction (Gao et al., 2016; Godzien et al., 2015). In addition, these approaches usually failed to comprehensively identify lipid species in each lipid class (Zheng et al., 2017).

Here, we developed an analytical method for comprehensive lipidomics analysis for plant glycerolipids by UPLC‐TripleTOF with MS/MS^ALL^ scan mode. Lipids in different classes were quantified by comparison with internal standards. This adapted MS/MS^ALL^ method for glycerolipids analysis exhibited advantages of high resolution, high sensitivity, high efficiency, and accurate *m/z*. It is easy to operate, and data analysis is simple. Previously, glycerolipids have not been comprehensively analyzed in seeds during different development stages of rapeseed (Katavic, Agrawal, Hajduch, Harris, & Thelen, 2006; Lu et al., 2016; Tang et al., 2012; Woodfield et al., 2017). We thus employed this approach to comprehensively analyze the glycerolipids from crude lipid extracts of rapeseed tissues in order to facilitate the understanding and study of lipid metabolism in rapeseed.

## 2 MATERIALS AND METHODS

### 2.1 Instruments and reagents

A LC‐30AD UPLC chromatography system (Shimadzu Corporation) coupled with TripleTOF 5600 (SCIEX) was used for injection and analysis of lipids. Analyst^R^ TF 1.6 and MultiQuant^TM^ data acquisition software (AB SCIEX), PeakView 2.0 (AB SCIEX) and LipidView 2.0 (AB SCIEX) data analysis software were used in experiment and data analysis. Two mobile phases A and B solution were prepared according to the formula water:methanol:acetonitrile:300 mM ammonium acetate = 20:20:20:1 (v/v/v/v) and isopropanol:methanol:300 mM ammonium acetate = 180:20:3 (v/v/v), respectively. Mobile phases A and B were sonicated for 30 min using an ultrasonicator before use. All reagents were HPLC grade, and the water was obtained from the Milli‐Q machine (Millipore).

### 2.2 Internal standards information

Internal standards of phospholipids and galactolipids were provided by Dr. Ruth Welti's laboratory at Kansas State University, USA. Dr. Ruth Welti's laboratory purchased the glycerolipid standards from Avanti Polar Lipids and Matreya, including phospholipids PC‐12:0/12:0, PA‐14:0/14:0, PE‐12:0/12:0, PG‐20:0/20:0, PI‐16:0/18:0, PS‐20:0/20:0, lysophospholipids LPC‐19:0, LPG‐18:0, LPE‐18:0, galactolipids MGDG‐18:0/18:0, and DGDG‐18:0/18:0. TAG‐17:0/17:0/17:0 was purchased from Sigma‐Aldrich, and DAG‐17:0/17:0‐d5 was purchase from Avanti Polar Lipids. Internal standards were weighted and quantified as fatty acid methyl esters by GC following the method described by Welti et al. (2002) before preparation of mixed internal standards. Five μl mixture containing 0.06 nmol/μl of PA, PE, PG, LPE, and LPG, 0.12 nmol/μl of PC and LPC, 0.04 nmol/μl of PS, 0.0574 nmol/μl of PI, 0.4 nmol/μl of TAG, 0.2 nmol/μl of DAG, 0.281 nmol/μl of MGDG, and 0.296 nmol/μl DGDG were combined to serve as internal standards to quantify the lipid content of extracts.

### 2.3 Plant growth condition, sample collection, and lipid extraction

Rapeseed grew in the field under natural condition. Root, stem, leaf, and petiole tissues were cut off from 60‐day‐old plant quickly and immersed into a glass tube with 75°C isopropanol immediately for lipid extraction as described previously (Zien, Wang, Wang, & Welti, 2001). Briefly, sample was heated in 3 ml 75°C isopropanol including 0.01% BHT in a glass tube A for 15 min, and then, the tube was set under room temperature for 10 min. A 1.5 ml chloroform and 0.6 ml water were added into the tube A, and the mixture was shaken for 60 min, and the extract was transferred into a new glass tube B. A 4 ml chloroform:methanol = 2:1 (v/v) mix containing 0.01% BHT was added into the tube A and shaken for 30 min, and the extract was transferred into the tube B again. The extraction was repeated 4–5 times until the color of the sample turned white. One ml 1 M KCl was added to the tube B and mixed, the mixture was centrifuged at 1,000 *g* for 5 min, and the supernatant was discarded. Subsequently, 2 ml ddH_2_O was added and mixed. The mixture was centrifuged at 1,000 *g* for 5 min, and the supernatant was discarded. Finally, the extract in tube B was dried by a stream of nitrogen and transferred to a 2‐ml sample bottle. The tissue in tube A was dried by an oven overnight, and the dry weight was measured. Dried lipid sample was diluted to 2 mg/ml based on dry weight was injected for lipid analysis. Flowering flowers were labeled, and the seeds were collected from 21, 26, 31, 36, 41, 46, 51, and 56 days after flowering (DAF) siliques and also immediately immersed into 75°C isopropanol for lipid extraction followed above lipid extraction method. 0.2–2 mg/ml based on dry weight working solution was diluted for lipid analysis.

### 2.4 UPLC conditions

Ten μl aforementioned diluted lipid extract was separated by the LC‐30AD UPLC with an Acquity UPLC^TM^ BEH C18 column (2.1 mm × 100 mm, i.d., 2 μm) using the mobile phases A and B solution. The temperature of column was set at 45°C. The flow rate of the eluent was 0.3 ml/min. The column was eluted with a linear gradient of 25% B over 1–2 min, 25%–40% B over 2–4 min, and 40%–95% B over 4–22 min, and 95% B was held for 5 min and then returned to 25% B to re‐equilibrate the column for 5 min before the next sample was injected. Fifty μl correction fluid (SCIEX, A7011) was injected every five samples to calibrate the mass spectrometer.

### 2.5 TripleTOF‐MS/MS conditions

The conditions of mass spectrometry were set as previously described with some modifications (Zheng et al., 2017). Briefly, mass spectrometry analysis was performed using a TripleTOF mass spectrometer (TripleTOF‐MS/MS) instrument with an electrospray ionization (ESI) source in positive ion mode. The source parameters were set as follows: ESI source temperature was set at 550°C. The voltage of ion spray and declustering potential was 5.5 kV and 100 V, respectively. The pressure of atomization gas and auxiliary air was both 50 psi, and nitrogen curtain gas was 35 psi. MS data were collected in TOF‐MS scan information‐dependent acquisition (IDA)‐fragmentation ion scan mode. The parameters of IDA filtered selective precursor ions were set as: The dynamic background subtraction was opened, and the top 10 most intense precursor ions with *m/z* of 400–1,000 Da and an intensity greater than 100 cps were selected as the selective criteria. For TOF‐MS scan, the *m/z* of 400–1,000 Da was set and the accumulation time was 250 ms. Fragmentation ion scans were set to an *m/z* range of 80–1,000 Da, and the accumulation time was 100 ms, and the collision energy was 35 ± 15 eV.

### 2.6 Data collection and analysis

The data were obtained using Analyst^R^ TF 1.6 and MultiQuant^TM^ data acquisition software. Data were processed using PeakView 2.0 software (Figure S1). The accurate molecular weight, mass‐to‐charge ratio (*m/z*), and fragmentation ion information were acquired with LipidView 2.0 to generate an in‐house dataset. The identification of glycerolipids classes and species was based on retention time, accurate *m/z*, and fragmentation ions patterns. The content of different lipids species was quantified by comparison with internal standards, and heat map was drawn by software ImageGP (http://www.ehbio.com/ImageGP/).

## 3 RESULTS

### 3.1 Experimental workflow for lipid analysis in rapeseed

**Figure 1:**
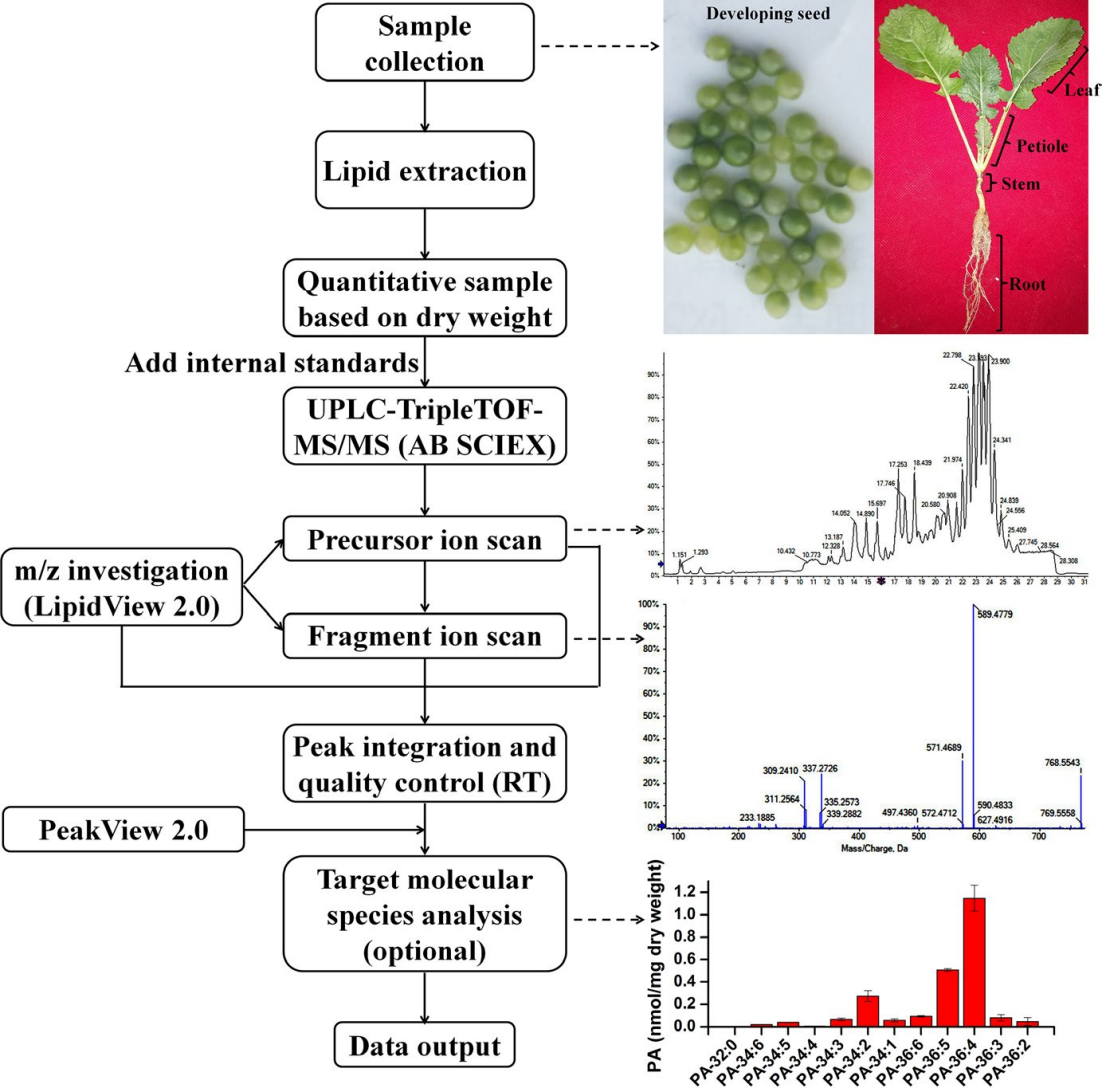
Experimental workflow for lipid analysis of different organs of *Brassica napus* with subsequent identification and quantification of different lipid classes and species using UPLC coupled to TripleTOF‐MS/MS. PA, phosphatidic acid; RT, retention time

Samples were collected from rapeseed plants in the field. Total lipids were extracted, and the dry weight of the residue was measured. Suitable sample concentration was diluted and mixed with a mixture of internal standards, and 10 μl sample was injected into UPLC coupled with TripleTOF. LC condition was optimized to allow separation of different lipid classes within 32 min. TOF‐MS scan collected lipid precursors and their product ions using the MS/MS^ALL^ based on data‐independent acquisition method. The accurate *m/z* of precursor and fragmentation ion of target lipids was investigated to generate a list of target lipids (Supplemental dataset) as in‐house database using LipidView according to previous reported lipid species in *Arabidopsis*, rapeseed, and other plants (Devaiah et al., 2006; Lu et al., 2018; Wei et al., 2016). Lipid species were identified and quantified by searching the target glycerolipid precursors from the collected MS/MS^ALL^ data of metabolites against the in‐house database using PeakView. The majority of lipid species can be accurately identified based on their accurate *m/z*. For lipid species with ambiguous *m/z* values, manual validation based on the retention time and fragmentation ions was performed (Figure 1).

### 3.2 Adduct pattern, characteristic fragment, identification fragmentation ion of different lipid class, and their *m/z* investigation

Thirteen glycerolipid classes including TAG, DAG, PC, PA, PE, PG, PI, PS, LysoPC, LysoPE, LysoPG, MGDG, and DGDG were investigated for adduct preference of precursor ion under positive ion mode using LipidView software. TAG, DAG, PA, PG, PI, MDGD, and DGDG formed primarily [M + NH_4_]^+^, and the rest of the lipids formed primarily [M + H]^+^ (Table S1). The *m/z* of characteristic fragment was also predicted by LipidView. All lipid classes except TAG and DAG were able to lose head group (HG) which can be used for validation of lipid class if necessary. Identification fragmentation ion was detected with the pattern of [M + H − NL]^+^ or [M + NH_4_ − NL]^+^ except TAG and DAG was the pattern of [M + NH_4_ − R]^+^ (R means one fatty acid chain), PC and LPC were [PRC + H]^+^ (PRC, phosphorylated choline). The *m/z* of precursor ion and identification fragmentation ion of all glycerolipid species were generated by LipidView (Supplemental dataset).

### 3.3 Separation and detection of lipids

**Figure 2:**
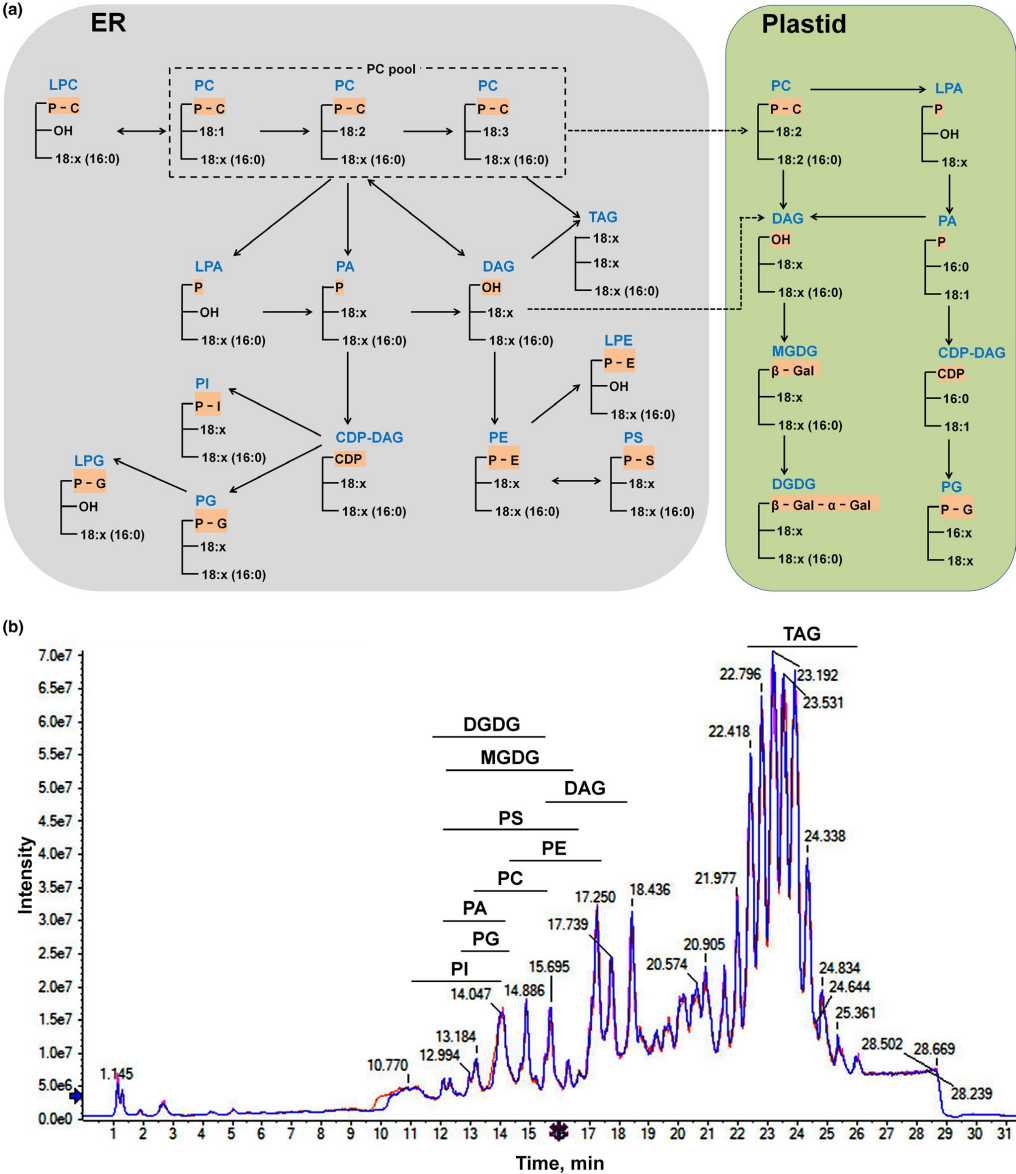
Glycerolipid biosynthesis pathway and total ions chromatogram of lipids. (a) Simplified glycerolipid biosynthesis pathway in ER and plastid modified from Acyl‐lipid metabolism (Li‐Beisson et al., 2013). (b) Total ions chromatogram from an Acquity UPLC^TM^ BEH C18 column and peak pattern of lipids. Three injections of the same sample extracted from the seeds. DAG, diacylglycerol; DGDG, digalactosyldiacylglycerol; ER, endoplasmic reticulum; MGDG, monogalactosyldiacylglycerol; PA, phosphatidic acid; PC, phosphatidylcholine; PE, phosphatidylethanolamine; PG, phosphatidylglycerol; PI, phosphatidylinositol; PS, phosphatidylserine; TAG, triacylglycerol

**Figure 3:**
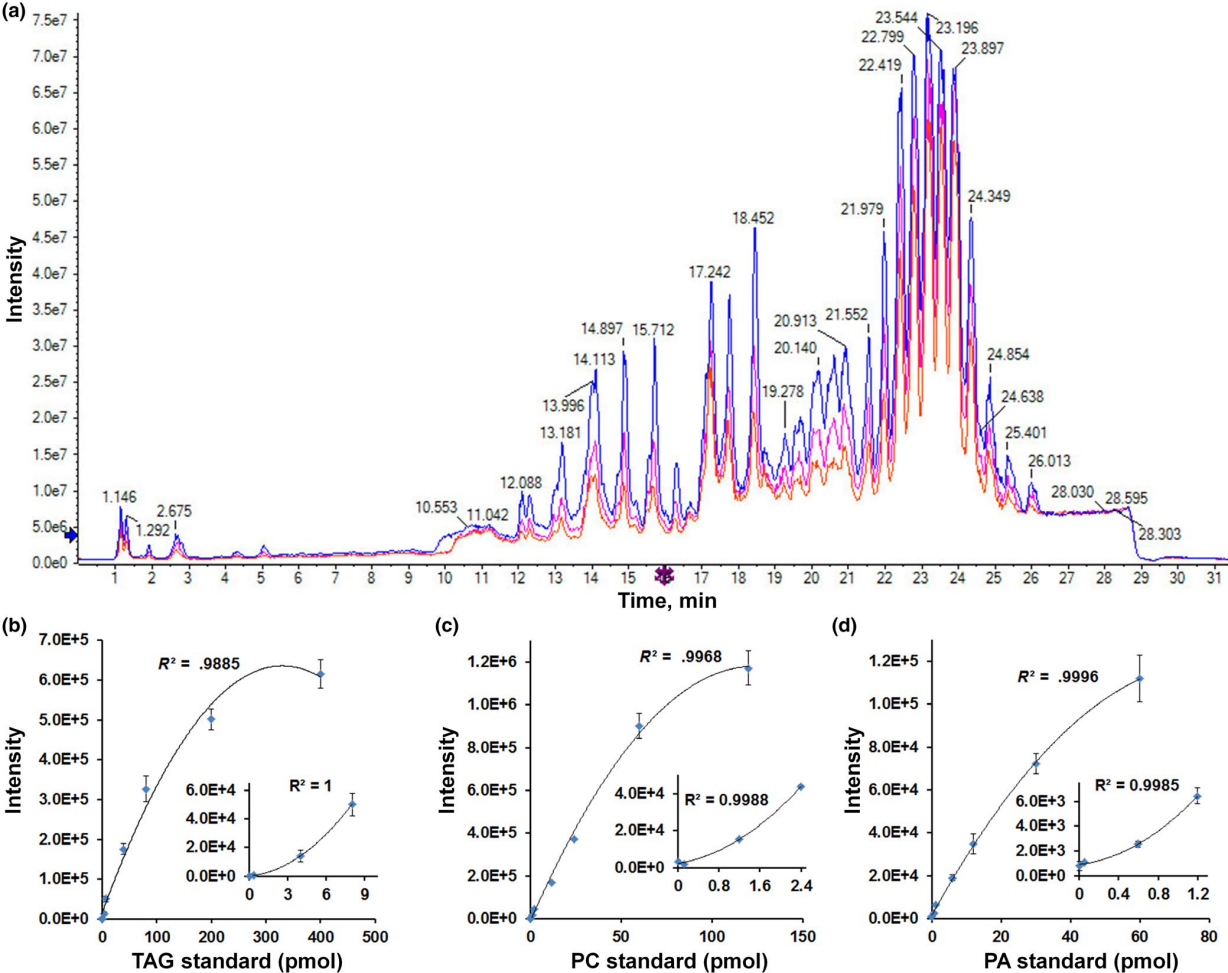
Comparison of total ions chromatogram of three concentrations of lipids and the validation of the linear range detection of lipids. (a) Total ions chromatogram of three concentration of the same sample extracted from seeds. Blue line, 2 mg dry weight/ml; pink line (the middle peak), 1 mg dry weight/ml; and red line (the lowest peak), 0.5 mg dry weight/ml. (b) Linear regression *R* ^2^ value was .9885 of TAG standard, over the range 0.04–400 pmol. Inset is the figure for the amplification of linear range over 0.04–8 pnmol. (c) Linear regression *R* ^2^ value was .9968 of PC standard, over the range 0.012–120 pnmol. Inset is the figure for the amplification of linear range over 0.012–2.4 pnmol. (d) Linear regression *R* ^2^ value was .9996 of PA standard, over the range 0.006–60 pnmol. Inset is the figure for the amplification of linear range over 0.006–1.2 pnmol. More linear regression *R* ^2^ value and linear range were shown in Figure S2. PA, phosphatidic acid; PC, phosphatidylcholine; TAG, triacylglycerol

We aimed to analyze glycerolipids including phospholipids, galactolipids, TAG, and DAG (Figure 2a). Total lipids extracted from the mature seeds of rapeseed were separated by the liquid chromatogram although some lipid classes overlapped with each other (Figure 2b). Galactolipids and phospholipids mainly eluted from the column between 11 and 17.5 min. PC was from 13.17 to 15.71 min. PA was from 12.13 to 14.18 min, and MGDG was from 12.09 to 16.56 min etc. DAG was eluted between 15.52 and 18.45 min, and TAG eluted last between 22.32 and 25.95 min. Three replicates of the same lipid extract were injected to investigate the instrumental repeatability and stability. Total ions chromatogram including peak pattern and retention time of three replicates overlapped well (Figure 2b). To test the instrumental response to different concentrations of lipids, three concentrations (0.5, 1, and 2 mg/ml based on dry weight) of the same lipid extract were injected into the instrument. The chromatogram displayed that the peak height of these three concentrations responded nicely, and the retention time of the same peak was consistent among different samples (Figure 3a). Together, these results indicate that UPLC coupled with TripleTOF had good repeatability, stability, and sensitivity for plant lipid detection.

### 3.4 Determination of the instrumental linear range and accuracy of lipid metabolites

In order to determine the linear range of the different lipids, 8 different concentrations of mixed internal standard containing 0.001‐, 0.01‐, 0.1‐, 0.2‐fold, onefold, twofold, fivefold, and 10‐fold to their working solution concentration (described in method) were spiked‐in the same concentration extracts diluted from one sample. A mixture containing 13 internal standards with the same dilution fold was injected into the instrument, three replicates for each mixture. The results showed that the concentration of TAG had great linear correlation between 0.4 and 80 pmol by linear regression analysis (Figure 3b; Figure S3). PC had linear correlation between 0.12 and 60 pnmol (Figure 3c; Figure S3) and PA had linear correlation between 0.06 and 30 pmol (Figure 3d; Figure S3). Other lipids also had linear correlation within certain concentration. For example, DAG was in 0.2–40 pmol, PE in 0.06–30 pmol, PG in 0.06–30 pmol, PI in 0.057–28.5 pmol, PS in 0.04–20 pmol, MGDG in 0.281–56.2 pmol, and DGDG in 0.296–59.2 pmol (Figures S2a‐g and S3). These data show that TripleTOF had broad linear range for most lipid classes which would guide the lipid sample preparation for TripleTOF analysis.

**Figure 4:**
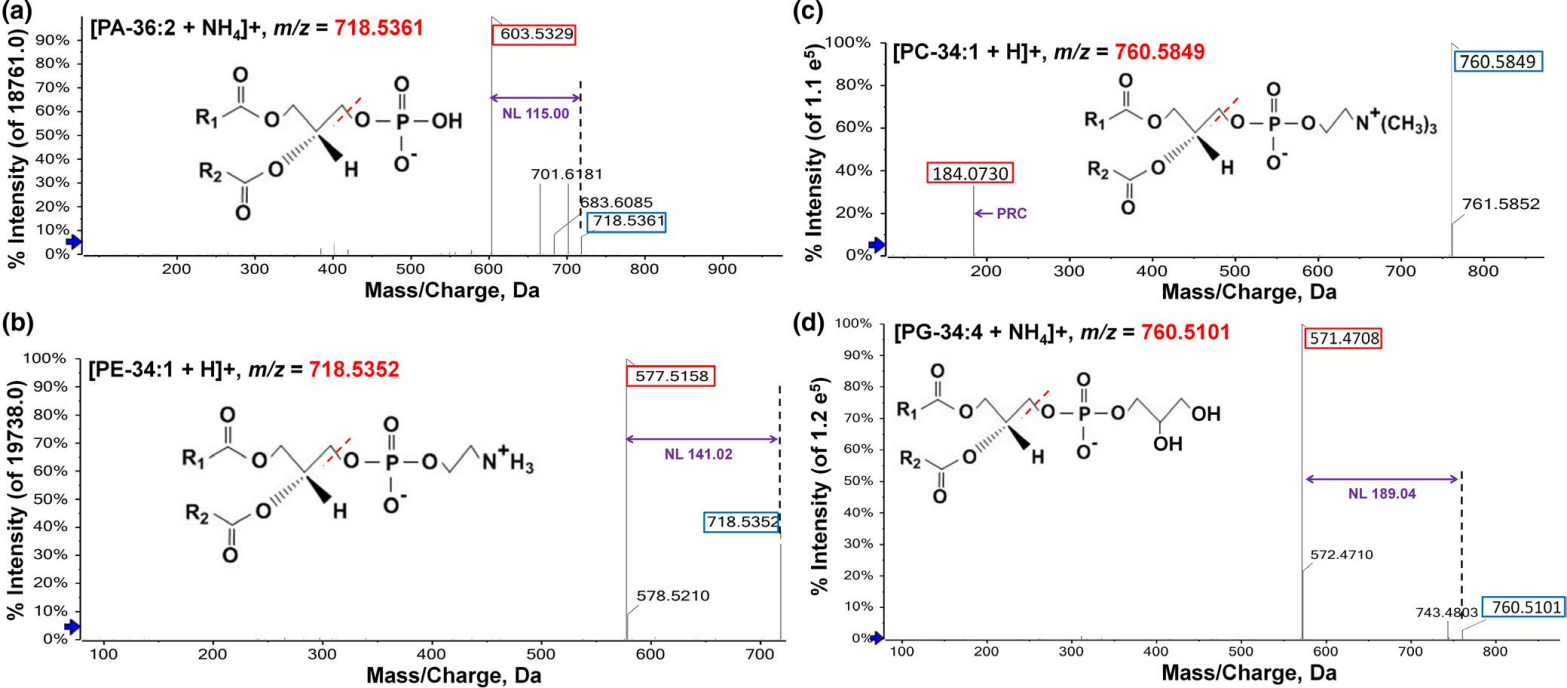
Comparison of representative precursor ions adducts with close *m/z* and their characteristic fragmentation ions. (a) and (b), the *m/z* was 718.5361 of PA‐36:2 and 718.5352 of PE‐34:1, respectively. Neutral loss of PA was 115.00 and PE was 141.02. (c) and (d), the *m/z* was 760.5849 of PC‐34:1 and 760.5101 of PG‐34:4, respectively. The *m/z* of head group of PC was 184.07 and neutral loss of PE was 189.04. The number in blue frame was the *m/z* of precursor ion adduct and red frame was the *m/z* of that product ion adduct. Purple number was the *m/z* of characteristic fragmentation ion adduct (neutral loss). NL, neutral loss

Mass accuracy is an important parameter for estimating the quality of the instrument. The *m/z* of different lipid species adducts and their characteristic fragmentation ions were predicted by LipidView, and 4 significant figures after the decimal point were predicted for each adduct (Supplemental dataset). The results showed that almost all the *m/z* of internal lipid standards detected by instrument were consistence with that from prediction at the level of 2 significant figures after the decimal point at least. The fragmentation ion also achieved similar accuracy (compare Figure S4 to dataset). Almost all target glycerolipid species could be differentiated and thus identified based on the accuracy of detected precursors. For example, the *m/z* of PC‐34:1 adduct is predicted to be 760.5851 and detected as 760.5849. The *m/z* of PG‐34:4 adduct was predicted to be 760.5123 and detected as 760.5101 (Figure 4c,d). The resolution of the machine for PC‐34:1 and PG‐34:4 was 0.26 and 2.89 ppm, respectively. PC‐34:1 and PG‐34:4 could be accurately identified based on their detected precursor *m/z.* In rare cases, if two different lipid species have extremely close *m/z*, they could be identified by looking at their characteristic product ions. For example, PA‐36:2 adduct had a detected *m/z* of 718.5361, while PE‐34:1 adduct was detected to be 718.5352 (Figure 4a,b). The resolution of the machine for PA‐36:2 and PE‐34:1 was 2.78 and 4.18 ppm, respectively. In fact, PA(36:2) [M + NH4]^+^ and PE(34:1) [M + H]^+^ have the same chemical formula (C_39_H_77_O_8_PN) and the same theoretical m/z. It was hard to distinguish these two pairs of species by judging the *m/z* of the precursor ion adduct. However, the fragmentation ion could be tracked to identify each species according to their *m/z* (Figure 4a,b). In addition, these two species could also be validated by their different retention time. The NL *m/z* of other lipids is showed in Table S1 and Figure S4. These results indicate that lipid species could be identified by accurate *m/z* of precursor ion adduct.

### 3.5 Analysis of lipidome changes in developing seed of rapeseed

**Figure 5:**
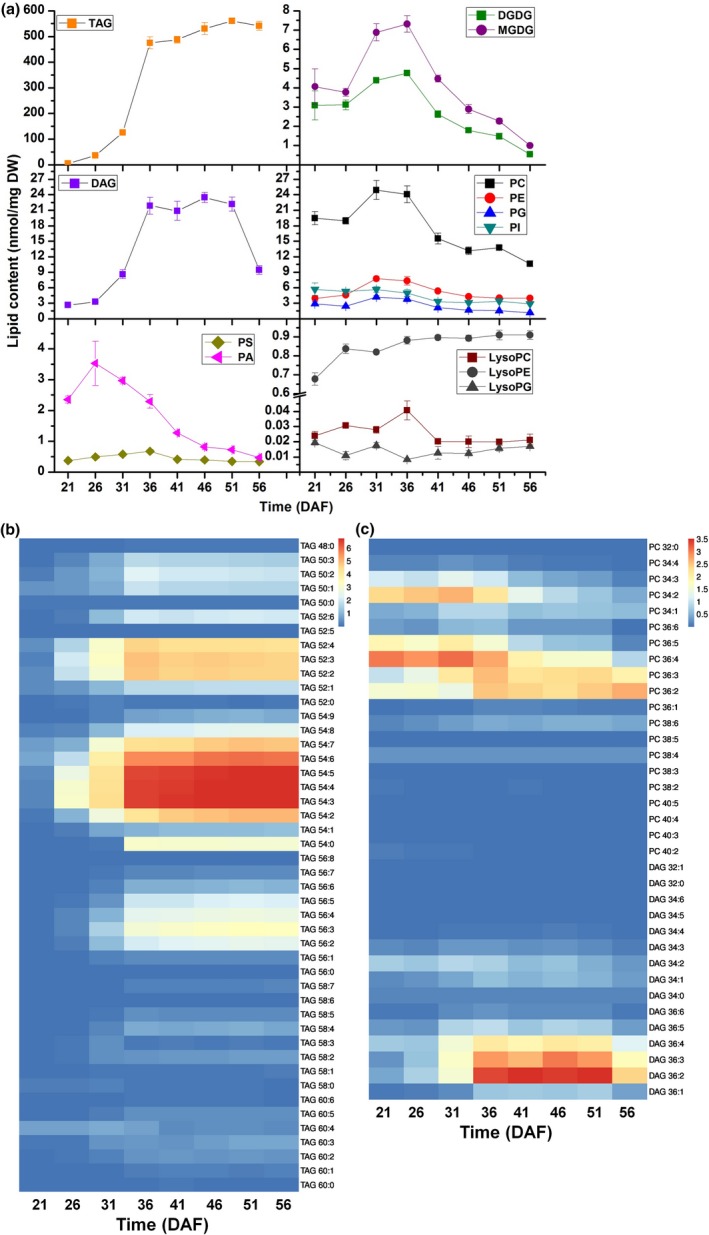
Lipid content and composition of developing seed of *Brassica napus*. (a) The content of all glycerolipids in 21, 26, 31, 36, 41, 46, 51, and 56 DAF seeds. Values are means ± *SD* (*n* = 5–6). (b) Content of different TAG species in 21, 26, 31, 36, 41, 46, 51, and 56 DAF seeds. (c) PC and DAG species content in 21, 26, 31, 36, 41, 46, 51, and 56 DAF seeds. Heat map was drawn by software ImageGP

In order to evaluate this approach, we analyzed the lipid composition and level in developing seeds of rapeseed. Total lipids of developing seeds collected at 21, 26, 31, 36, 41, 46, 51, and 56 DAF were extracted with 5–6 replicates for each stage. Diluted samples mixed with internal standards were analyzed by UPLC‐TripleTOF, and lipid metabolites were scanned with MS/MS^ALL^ based on data‐independent acquisition method. For each analysis, over 2,000 metabolites could be scanned in crude lipid extract and data mining can be performed afterward. Target lipid species were identified and quantified by searching the MS/MS^ALL^ data against the in‐house lipid target dataset (Supplemental dataset). The level for each lipid class was calculated based on all lipid species in each class. The content of TAG accumulated quickly from 21 DAF to 51 DAF and then reduced slightly after 51 DAF (Figure 5a). TAG‐54:2 to TAG‐54:7 and TAG‐52:2 to TAG‐52:4 were the main TAG species in the seed in different development stages, and they accumulated quickly starting at 26 DAF (Figure 5b). Galactolipids MGDG and DGDG had a consistent trend in content change that both of them increased before 36 DAF and decreased after 36 DAF. The content of MGDG and DGDG was 7.32 and 4.76 nmol/mg at 36 DAF, respectively (Figure 5a). The content of MGDG‐34:6 and DGDG‐36:6 was the major species (Figure S5c). The trend of DAG content was observed like a bell shape, and it reached the highest content at 46 DAF (Figure 5a). The species of DAG‐36:2, ‐36:3, and ‐36:4 were the main DAG species (Figure 5c).

The trend of phospholipid including PC, PE, PG, and PI showed similar pattern as that of galactolipid (Figure 5a). The highest level of PC was 24.91 nmol/mg dry seed at 31 DAF with PC‐34:2, ‐36:2, ‐36:3, ‐36:4, and ‐36:5 as the major species (Figure 5c). The content of PA reached the highest level at 26 DAF and then decreased progressively (Figure 5a; Figure S3a). The level of lysolipids was also analyzed during different seed development stages (Figure 5a; Figure S5a,b). Collectively, determination of glycerolipids indicates dynamic changes in different lipid class/species during seed development in rapeseed.

### 3.6 Lipidome in root, stem, leaf, and petiole

**Figure 6:**
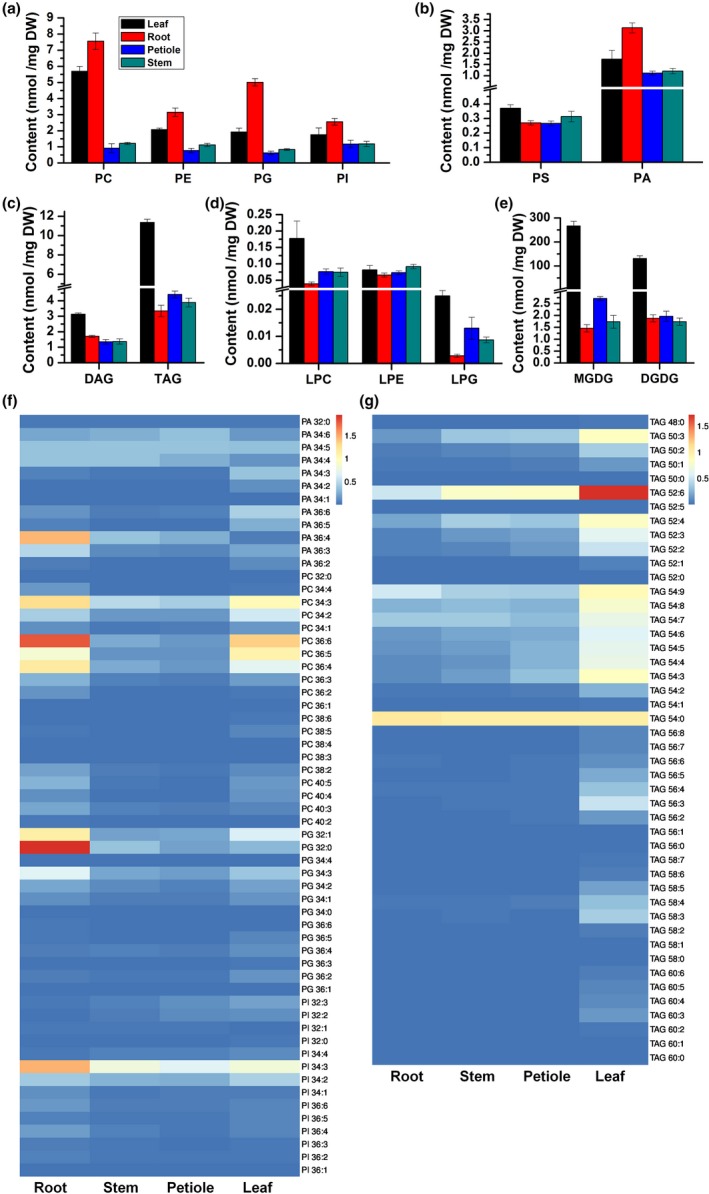
Lipid content and composition in root, stem, leaf, and petiole of 60‐day‐old *Brassica napus* plant in the field. (a) to (e) The content of glycerolipid in root, stem, leaf, and petiole of 60‐day‐old plant in the field. PC, PE, PG, and PI contents were showed in (a), PS and PA in (b), DAG and TAG in (c), LPC, LPE, and LPG in (d), MGDG and DGDG in (e). Values are means ± *SD* (*n* = 5–6). (f) The content of PA, PC, PG, and PI species in different tissues. (g) The content of TAG species in different tissues. Heat map was drawn by software ImageGP

Lipids were extracted from root, stem, leaf, and petiole of 60‐day‐old plants grown in the field, and glycerolipids were analyzed using the established approach. The results showed that the content of different lipid classes and species varied among different tissues. The content of MGDG, DGDG, DAG, TAG, PS, LysoPC, and LysoPG was highest in the leaf, while the content of PC, PE, PG, PI, and PA was highest in the root (Figure 6a‐e). The content of major phospholipids including PC, PE, PG, PI, and PA was significantly higher in leaf and root than that in petiole and stem. For example, PC content was 5.70, 7.55, 0.92, and 1.21 nmol/mg in leaf, root, petiole, and stem, and PE was 2.07, 3.15, 0.77 and 1.12 nmol/mg (Figure 6a,b). There was approximate 0.3 nmol/mg of PS in different tissues (Figure 6b). TAG had highest content in the leaf with 11.36 nmol/mg, while 3.34, 4.40, and 3.88 nmol/mg in root, petiole, and stem. DAG content was 3.13, 1.70, 1.35, and 1.37 nmol/mg in leaf, root, petiole, and stem (Figure 6c). The content of lysophospholipids was extremely low in all tissues but our approach successfully detected the major species (Figure 6d; Figure S6b,c). MGDG and DGDG are the major lipids of chloroplast membrane. The content of MGDG and DGDG was 266.82 and 131.22 nmol/mg in leaf, which was much higher than that of other three tissues (Figure 6e).

Many major lipid species had different content in different tissues (Figure 6f,g; Figure S6a–d). For example, the main species of TAG were C‐50:3, C‐52:6, and C‐52:2 to C‐52:4, C‐54:3 to C‐54:9, and C54:0 in leaf, and TAG‐52:6 was the highest species (Figure 6g). TAG‐52:6 and TAG‐54:0 were the highest species both in stem and petiole, and only TAG‐54:0 was the highest species in root (Figure 6g). Interestingly, the content of TAG‐54:0 had no difference in four tissues (Figure 6g). The content of other lipid species in different tissues was compared and shown in Figure 6f,g and Figure S6a–d. The results indicate that TripleTOF‐based lipidomics approach is suitable for analyzing lipids extracted from different plant tissues.

## 4 DISCUSSION

The analysis platform of the mass spectrometry represented by ESI‐MS/MS is widely used in the composition analysis of various biological tissue extracts (Bilgin et al., 2016; Cheong et al., 2014; Dennis, 2009; Han & Gross, 2005; Han et al., 2006; Horn & Chapman, 2012; Merrill, Sullards, Allegood, Kelly, & Wang, 2005; Shiva et al., 2013; Welti et al., 2002). Methods using neutral loss/precursor scan or multiple reaction monitoring (MRM) data collection are favored in the analysis of lipidome for their excellent performance in reliability and sensitivity (Gao et al., 2016; Simons et al., 2012). However, instrument operation requires careful optimization, multiple sample injections, and undesirable running time to achieve comprehensive analysis of phospholipids, galactolipids, and non‐polar TAGs/DAGs (Groessl et al., 2014; Welti et al., 2002). Recently, new mass spectrometry instruments are available with improved instrumental performance, especially in sensitivity, speed, and accuracy (Gao et al., 2018; Li et al., 2017; Zheng et al., 2017). For example, lower concentration of phospholipids in peanut can be identified by HILIC‐ESI‐IT‐TOF‐MS/MS system (Song et al., 2018). Species of phosphatidylglycerol can be quantified in 6 min using QTOF‐MS/MS (Gao et al., 2018). Many kinds of glycerolipids were identified in yellow sarson seeds in a short time based on a positive mode of ESI of the UPLC‐TripleTOF (Zheng et al., 2017). New hybrid instrument platform such as UPLC‐TripleTOF makes the lipid analysis more efficient and accurate.

In this study, we developed a lipid analysis method using UPLC‐TripleTOF with positive mode of ESI and MS/MS^ALL^ scan for data collection. Glycerolipids were separated using UPLC to reduce ion suppression which could hamper accurate detection of lipids (Tarazona et al., 2015). A target method dataset was generated by an information‐dependent acquisition (IDA) method with the LipidView software, and internal standards were designated to correct the specific lipid species (Supplemental dataset). The target method dataset was uploaded to the PeakView software to create processing datasets to search lipid species in collected data. This approach achieved the batch processing of multiple samples in lipid class‐specific screening experiments. Thirteen classes of glycerolipids containing 218 kinds of species were identified and quantified in 10 μl lipid solvent, including 46 TAGs, 15 DAGs, 20 PCs, 24 PEs, 13 PGs, 14 PIs, 26 PSs, 12 PAs, 16 MGDGs, 16 DGDGs, 6 LysoPCs, 5 LysoPEs, and 5 LysoPGs. Compared with the traditional shotgun approach based on ESI‐MS/MS, much less lipid solvent was required for phospholipid and galactolipid analyses (Groessl et al., 2014; Welti et al., 2002). In addition, our approach achieved comprehensive lipid profiling of 13 lipid classes by one injection in short time. Although Zheng et al. (2017) identified 144 glycerolipids species including 77 TAGs, 32 DAGs, 18 SQDGs, 5 MGDG, and 12 DGDGs with the UPLC‐TripleTOF in 22 min, the class of glycerolipids was limited and the absolute value of each species was not obtained due to lack of internal standards.

Lipid extracts from root, stem, leaf, petiole, and developing seed were analyzed in this study. The results were more comprehensive than that reported previously (Song et al., 2018; Zheng et al., 2017). Rapeseed lipid composition is comparable to *Arabidopsis*. For example, the content of PA‐36, PC‐36 and PI‐34 was higher than that of PA‐34, PC‐34, and PI‐36 in *Arabidopsis* seed (Devaiah et al., 2006). Similar patterns were also observed in rapeseed (Figure 5f; Figure S5a). The content of PS with >36 acyl carbons was the highest species in each tissue (Devaiah et al., 2006), and our results showed PS‐42:1 was the highest PS species in 4 rapeseed tissues (Figure S6b). The content of TAG‐54:3, TAG‐54:4, TAG‐54:5, PC‐36:2, and PC‐36:3 was the most abundance species in TAG and PC in mature rapeseed detected by MALDI‐MS and ESI‐MS/MS (Lu et al., 2018). These data were consistent with previous reported results that TAG‐54:3, TAG‐54:4, and TAG‐54:5 were the highest content species in TAG in 56 DAF seed. PC‐36:2 was the highest species in PC in 56 DAF seed (Figure 5b,c). The evidence above indicates that the results of this approach are reliable. However, this approach based on UPLC‐TripleTOF cannot distinguish the precise *sn*‐ position of fatty acid chain in TAG yet, which can be done with specific analysis approach by ESI‐MS/MS (Li, Baughman, et al., 2014; Li, Butka, et al., 2014). In this study, we only used one internal standard for quantification of each lipid class and we have not established a correction curve using 2 internal standards described by previously study (Welti et al., 2002). It would be ideal to use 2 internal standards with short and long acyl chains to establish a correction curve for each lipid class, which will provide better quantification results.

The analytical time for each sample could also be reduced. This current method ran about 32 min for one sample, and however, there were no lipids eluted during 3–10 min (Figure 2). In future, optimization of UPLC conditions such as column flow rate and mobile phase would reduce the analysis time. In our analysis, each MS/MS^ALL^ scan could detect more than 2,000 metabolites with precursors and their product ions and most of them could be lipids. The collected data can be further mined for interesting target lipids. Theoretically, this strategy can be expanded to analyze more plant lipid classes such as sphingolipids, cardiolipin, sterol lipids, and so on. In future, we can improve the lipid extraction method to include more kinds of lipids and develop a comprehensive target method for analysis of more lipid species. Taken together, we developed a rapid, efficient, and reliable approach based on UPLC‐TripleTOF. These results show that UPLC‐TripleTOF was sensitive and accurate for detection of both abundant and low abundant lipid species, which is a powerful tool for lipidomics analysis of plants.

## CONFLICT OF INTEREST

The authors declare no conflict of interest.

## AUTHOR CONTRIBUTIONS

L. Guo conceived the study. S. Lu, Q. Li, and C. Jin performed the sample collection and preparation. H. Liu and Q. Li analyzed the samples by LC‐MS/MS. S. Lu analyzed the data and wrote the manuscript. L. Guo and S. Lu revised the manuscript. All authors read and approved the manuscript.
