# Supplementary material for: An efficient and comprehensive plant glycerolipids analysis approach based on high‐performance liquid chromatography–quadrupole time‐of‐flight mass spectrometer
Source: Plant Direct. 2019 Nov 15;3(11):e00183. doi: 10.1002/pld3.183 (PMC6858605; doi:10.1002/pld3.183)
Supplement: Supplementary file 1 [file PLD3-3-e00183-s001.pdf]

TABLE S1. Characteristic fragment *m/z* and identification fragmentation ions of different lipid classes under positive ion mode of ESI.

Lipid class	Precursor ion pattern (ESI^+)	Characteristic fragment *m/z* (ESI^+)	Identification fragmentation ion (ESI^+)
TAG	$[M + NH_4]^+$	-	$[M + NH_4 - R]^+$
DAG	$[M + NH_4]^+$	-	$[M + NH_4 - R]^+$
PC	$[M + H]^+$	PRC 184.07	$[PRC + H]^+$
PA	$[M + NH_4]^+$	NL 115.00	$[M + NH_4 - NL]^+$
PE	$[M + H]^+$	NL 141.02	$[M + H - NL]^+$
PG	$[M + NH_4]^+$	NL 189.04	$[M + NH_4 - NL]^+$
PI	$[M + NH_4]^+$	NL 277.06	$[M + NH_4 - NL]^+$
PS	$[M + H]^+$	NL 185.01	$[M + H - NL]^+$
MGDG	$[M + NH_4]^+$	NL 179.08	$[M + NH_4 - NL]^+$
DGDG	$[M + NH_4]^+$	NL 341.13	$[M + NH_4 - NL]^+$
LysoPC	$[M + H]^+$	PRC 184.07	$[M + H - RPC]^+$
LysoPE	$[M + H]^+$	NL 141.02	$[M + H - NL]^+$
LysoPG	$[M + H]^+$	NL 172.02	$[M + H - NL]^+$

NL, neutral loss; R, any chain of multiple fatty acid chains; PRC, phosphorylated choline.
