# Supplementary material for: An efficient and comprehensive plant glycerolipids analysis approach based on high‐performance liquid chromatography–quadrupole time‐of‐flight mass spectrometer
Source: Plant Direct. 2019 Nov 15;3(11):e00183. doi: 10.1002/pld3.183 (PMC6858605; doi:10.1002/pld3.183)
Supplement: Supplementary file 2 [file PLD3-3-e00183-s002.pdf]

Open Peakview 2.0 software

↓

Open target file，then click “masterview” and choose “new session”

↓

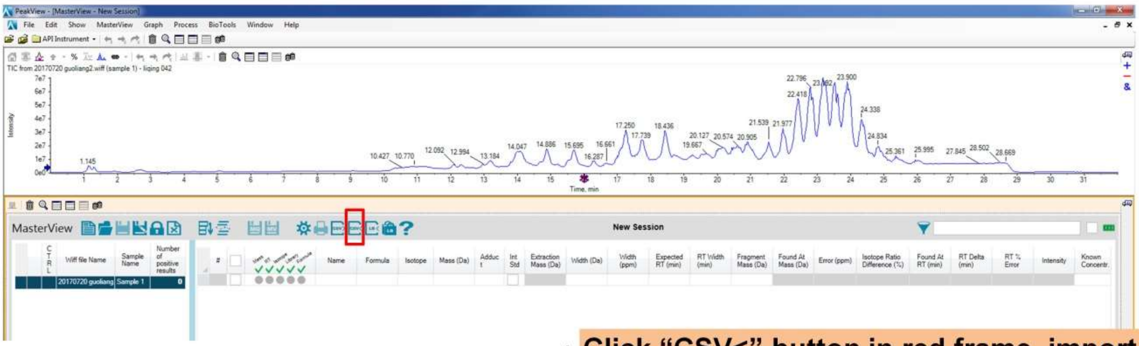

↓ Click “CSV<” button in red frame, import the supplementary dataset collected from Lipidview 2.0

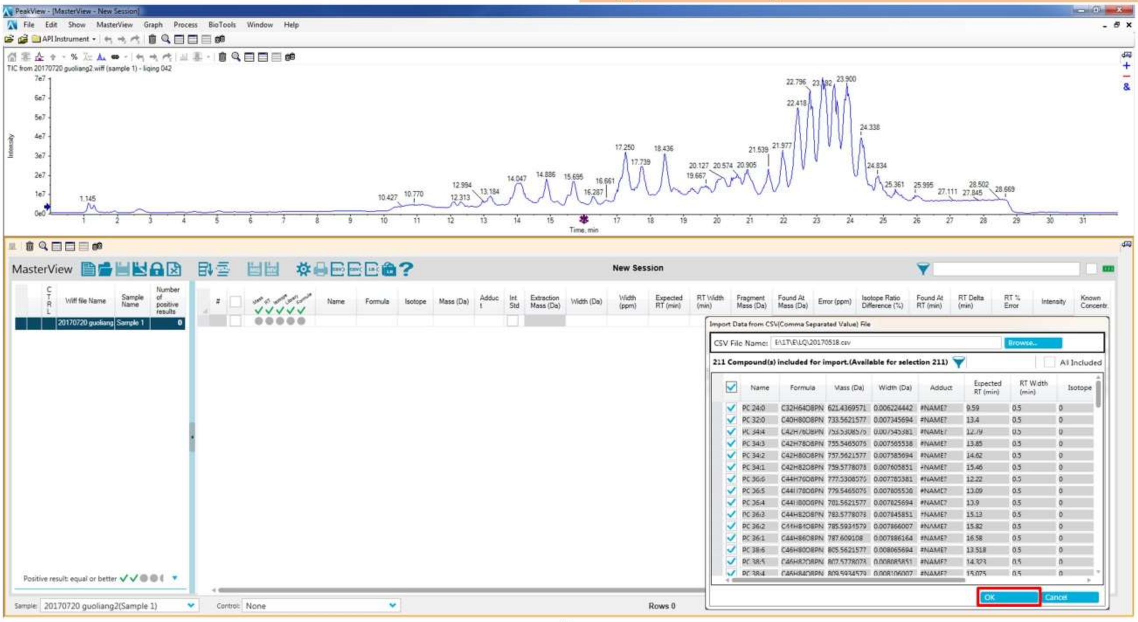

↓ “√” target species, click “Ok” button in red frame

Continue to next page

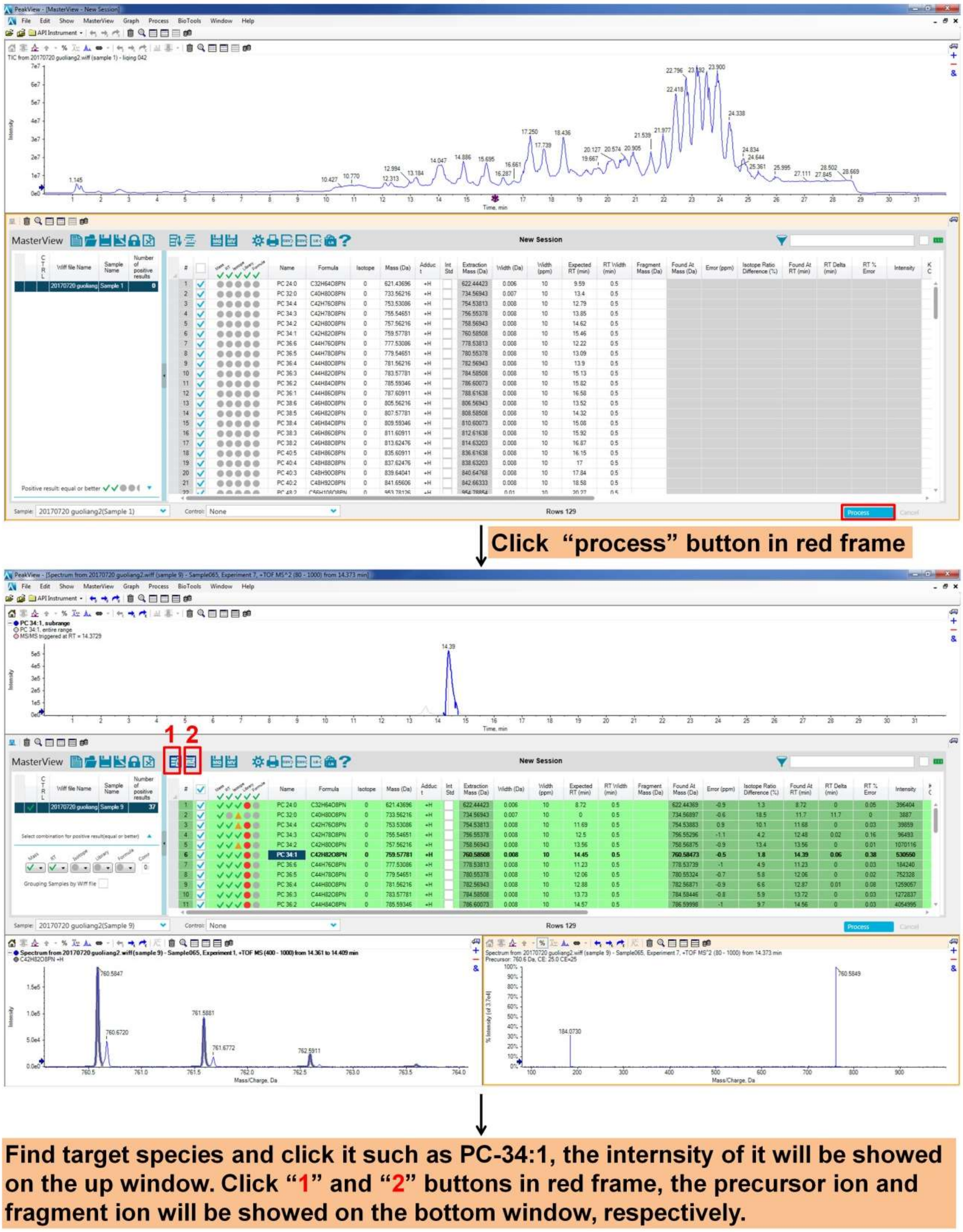

FIGURE S1. The workflow of data processing steps using PeakView 2.0 software.

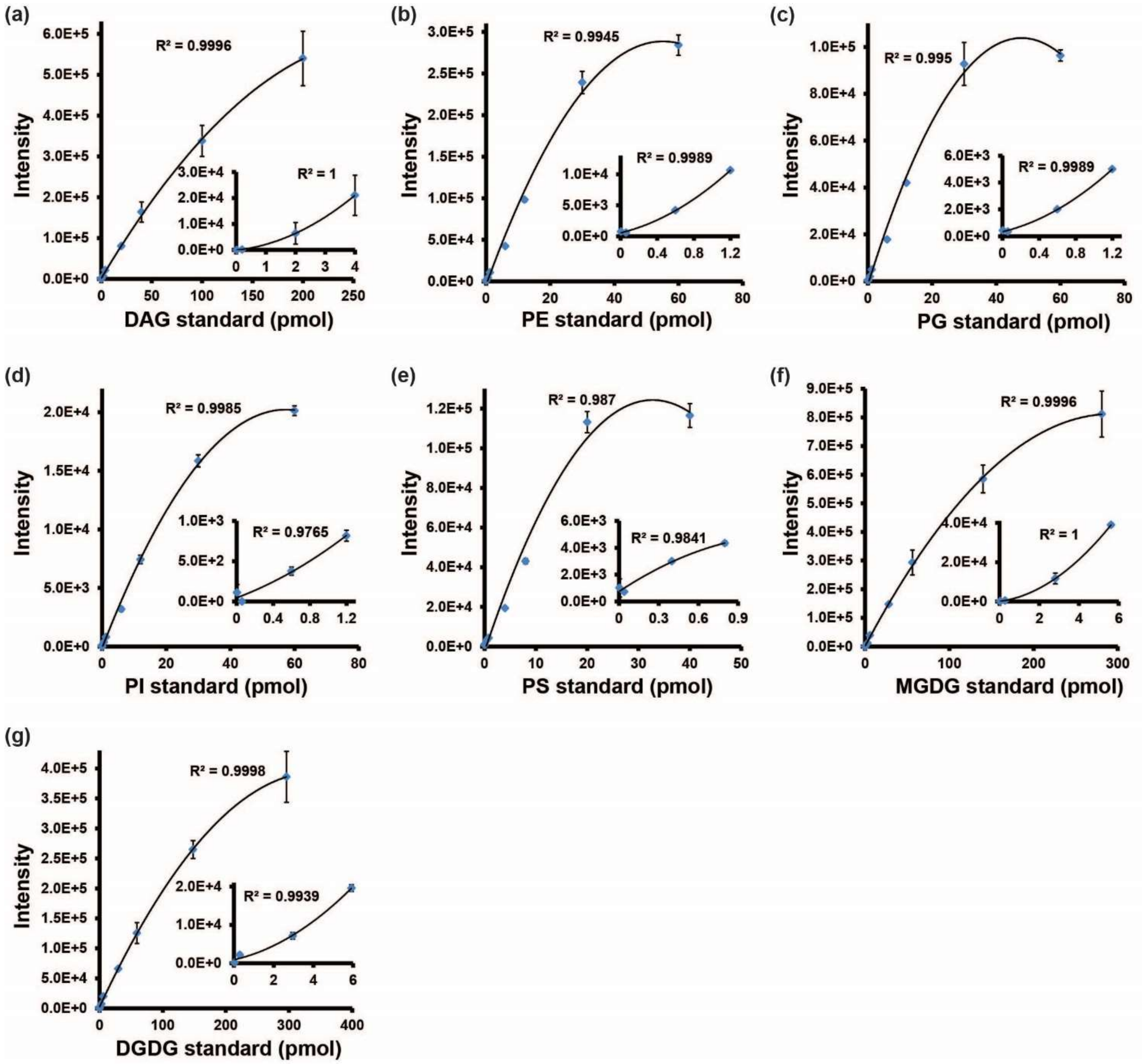

FIGURE S2. Validation of the linear-range was detected of internal standards. (a) Linear regression R^2 value was 0.9996 of DAG standard, over the range 0.02-200 pnmol. Inset shows the linear-range over 0.02-4 pmol. (b) Linear regression R^2 value was 0.9945 of PE standard, over the range 0.006-60 pmoll. Inset shows the the linear-range over 0.006-1.2 pmol. (c) Linear regression R^2 value was 0.995 of PG standard, over the range 0.006-60 pmol. Inset shows the linear-range over 0.006-1.2 pmol. (d) Linear regression R^2 value was 0.9985 of PI standard, over the range 0.0057-57 pmol. Inset shows the linear-range over 0.0057-1.15 pmol. (e) Linear regression R^2 value was 0.987 of PS standard, over the range 0.004-40 pmol. Inset shows the linear-range over 0.004-0.8 pmol. (f) Linear regression R^2 value was 0.9996 of MGDG standard, over the range 0.0281-281 pmol. Inset shows the linear-range over 0.0281-5.62 pmol. (g) Linear regression R^2 value was 0.9998 of DGDG standard, over the range 0.0296-296 pmol. Inset shows the linear-range over 0.0296-5.92 pmol.

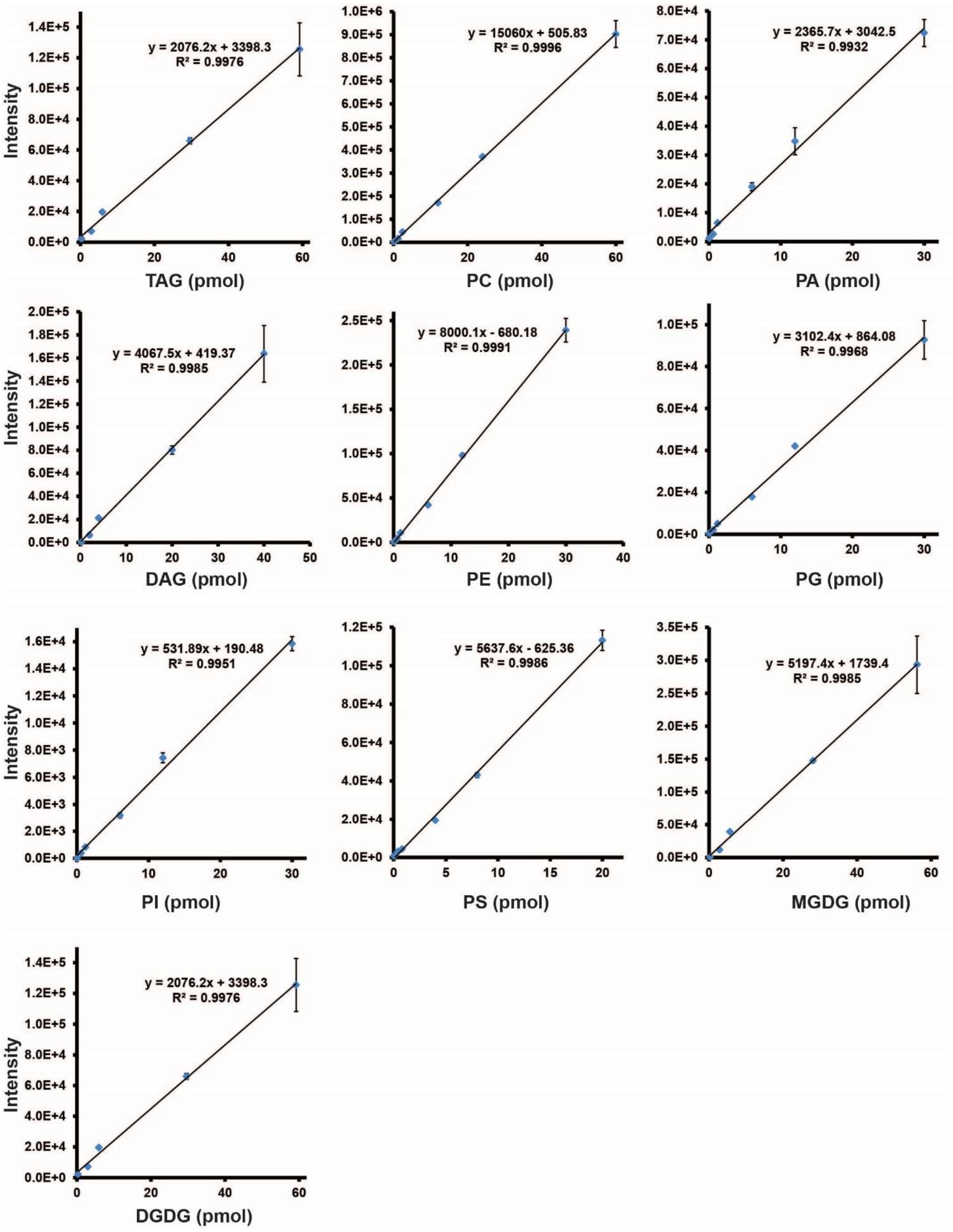

FIGURE S3. The linear range of instrument response to the internal standard concentration.

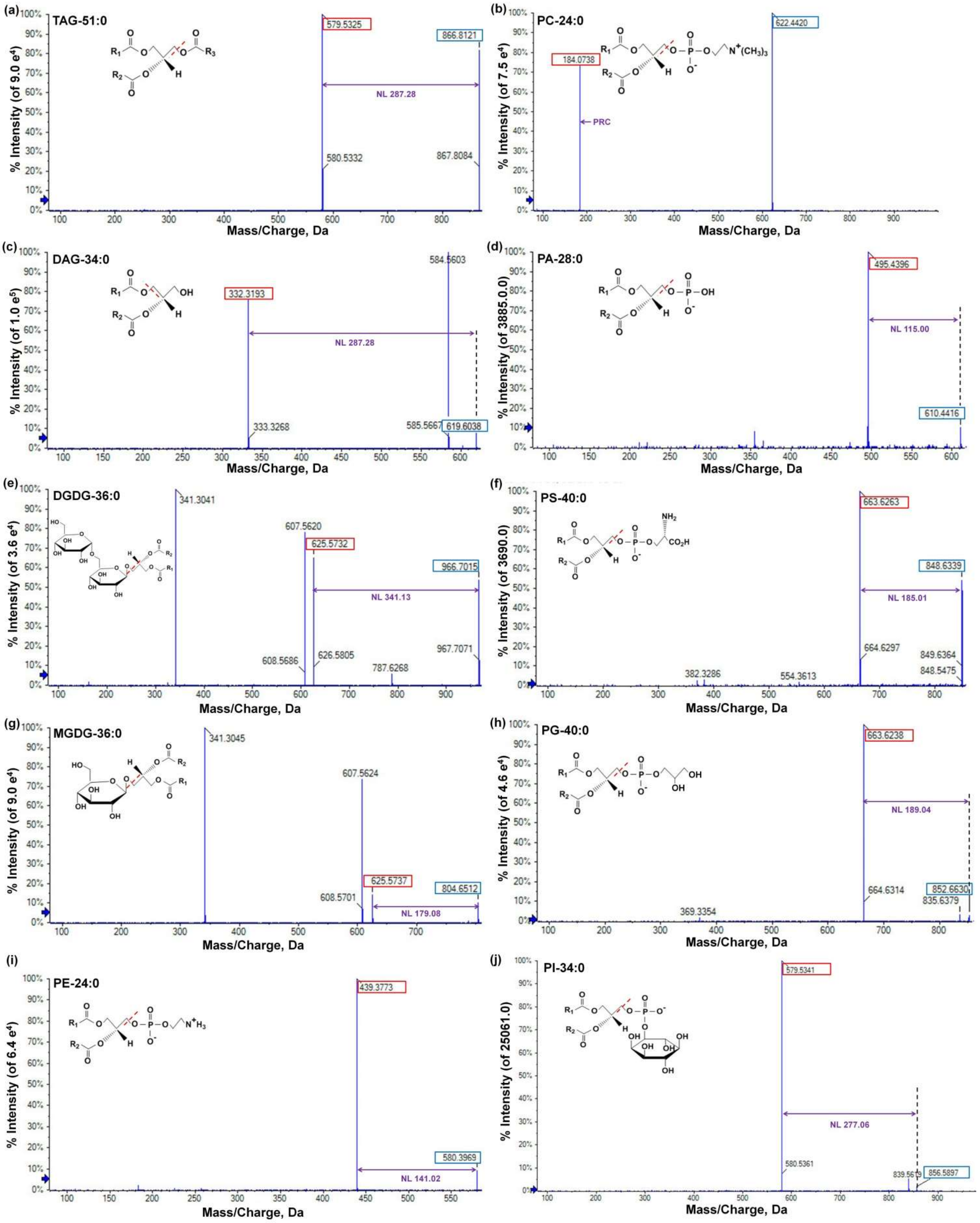

FIGURE S4. The *m/z* of precursor ion adduct and product ion adduct of internal standard lipid. (a) to (j) The *m/z* of precursor ion adduct and product ion adduct of TAG, PC, DAG, PA, DGDG, PS, MGDG, PG, PE, and PI standards. The number in blue frame represents the *m/z* of precursor ion adduct, red frame represents the *m/z* of that product ion adduct. Purple number represents the *m/z* of characteristic fragmentation ion adduct (neutral loss). NL, neutral loss. PRC, phosphorylated choline.

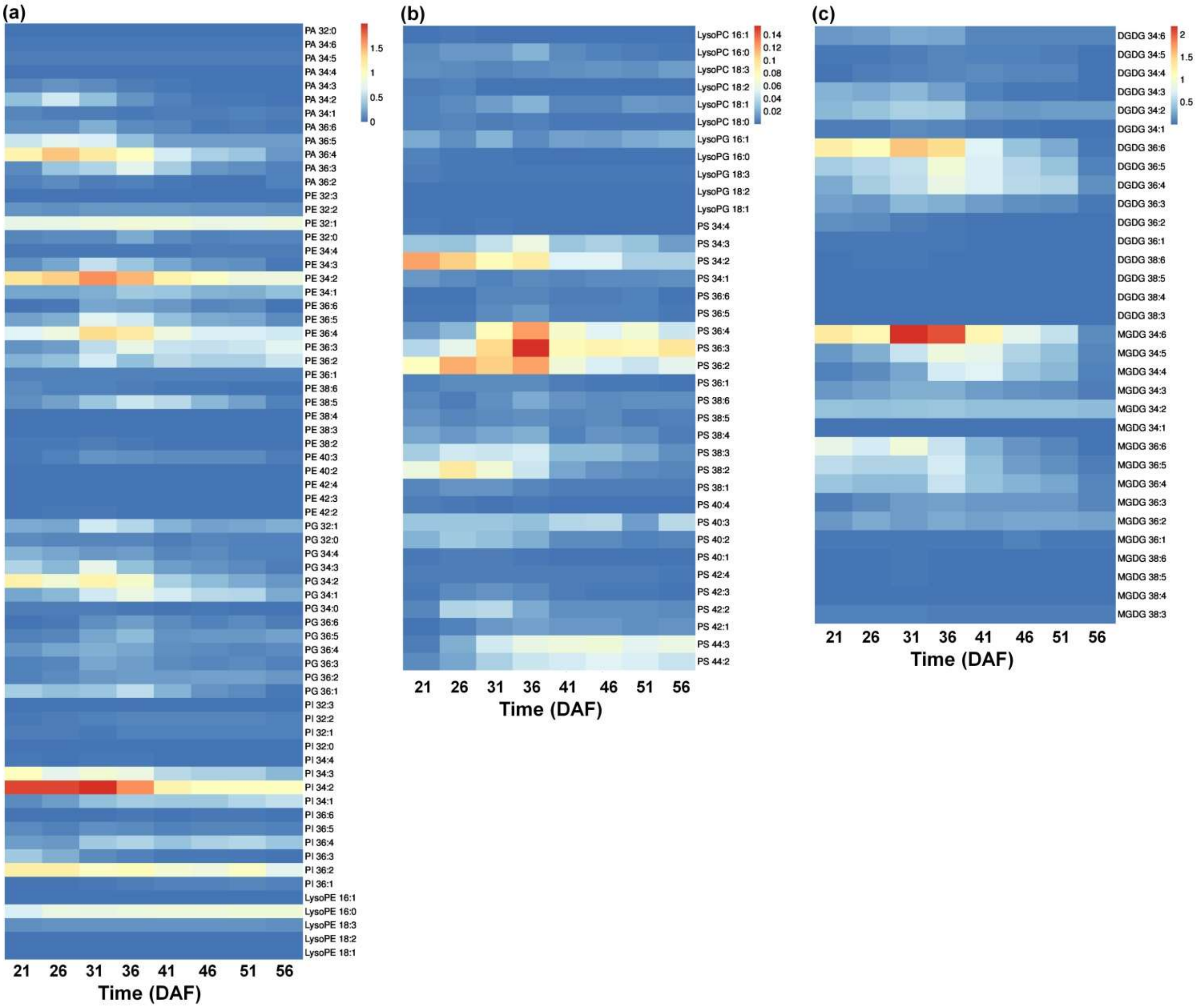

FIGURE S5. Lipid species quantified in 21, 26, 31, 36, 41, 46, 51, 56 DAF seeds of *Brassica napus*. (a) PA, PE, PG, PI, and lysoPE species. (b) LysoPC, lysoPG, and PS species. (c) DGDG and MGDG species. Heatmap was drawn by software ImageGP.

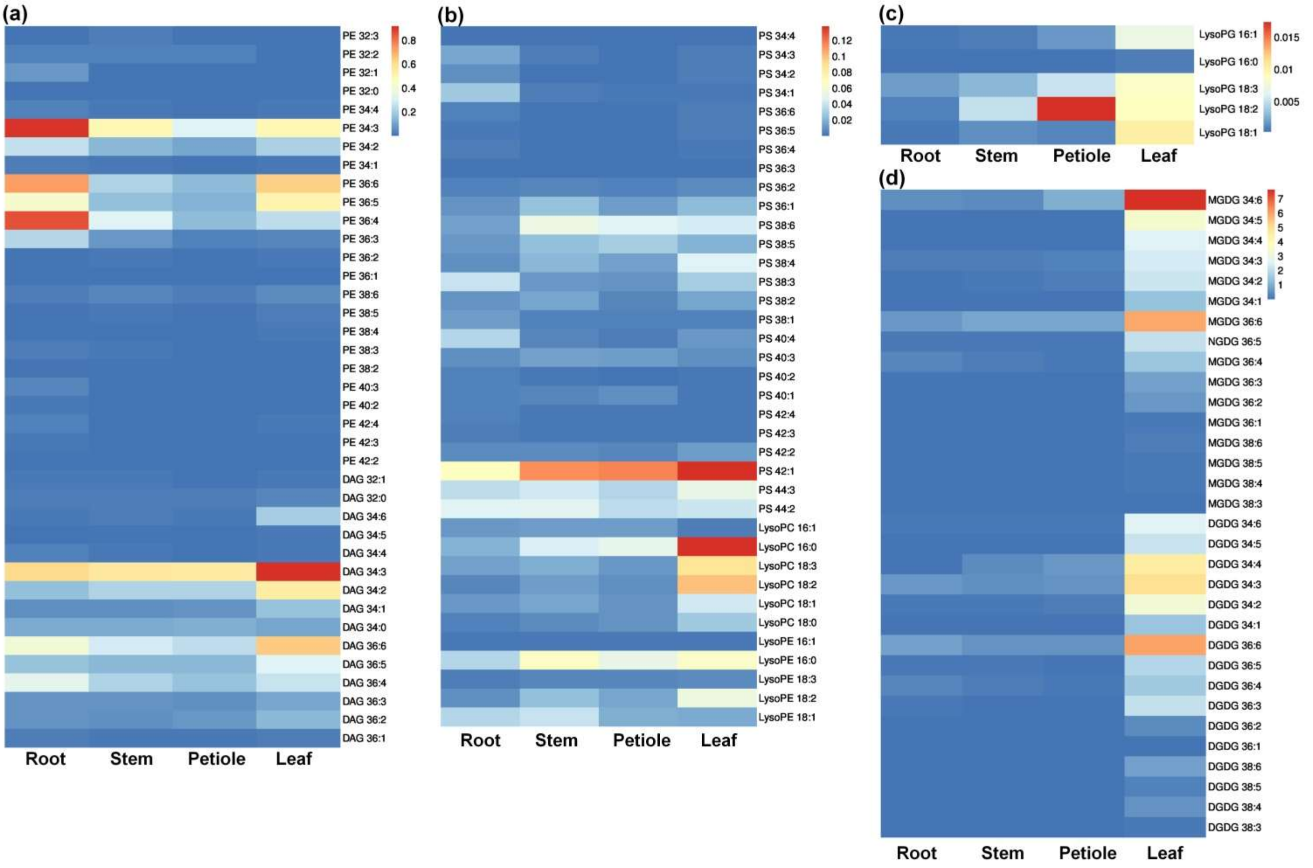

FIGURE S6. Lipid species quantified in root, stem, leaf, and petiole of 60-day old plant in the field. (a) PE and DAG species. (b) PS, lysoPC, and lysoPE species. (c) LysoPG species. (d) MGDG and DGDG species. Heatmap was drawn by software ImageGP.

TABLE S1. Characteristic fragment *m/z* and identification fragmentation ions of different lipid classes under positive ion mode of ESI.

Lipid class	Precursor ion pattern (ESI$^+$)	Characteristic fragment *m/z* (ESI$^+$)	Identification fragmentation ion (ESI$^+$)
TAG	$[M + NH_4]^+$	-	$[M + NH_4 - R]^+$
DAG	$[M + NH_4]^+$	-	$[M + NH_4 - R]^+$
PC	$[M + H]^+$	PRC 184.07	$[PRC + H]^+$
PA	$[M + NH_4]^+$	NL 115.00	$[M + NH_4 - NL]^+$
PE	$[M + H]^+$	NL 141.02	$[M + H - NL]^+$
PG	$[M + NH_4]^+$	NL 189.04	$[M + NH_4 - NL]^+$
PI	$[M + NH_4]^+$	NL 277.06	$[M + NH_4 - NL]^+$
PS	$[M + H]^+$	NL 185.01	$[M + H - NL]^+$
MGDG	$[M + NH_4]^+$	NL 179.08	$[M + NH_4 - NL]^+$
DGDG	$[M + NH_4]^+$	NL 341.13	$[M + NH_4 - NL]^+$
LysoPC	$[M + H]^+$	PRC 184.07	$[RPC + H]^+$
LysoPE	$[M + H]^+$	NL 141.02	$[M + H - NL]^+$
LysoPG	$[M + H]^+$	NL 172.02	$[M + H - NL]^+$

NL, neutral loss; R, any chain of multiple fatty acid chains; PRC, phosphorylated choline.
