# Supplementary material for: An efficient and comprehensive plant glycerolipids analysis approach based on high‐performance liquid chromatography–quadrupole time‐of‐flight mass spectrometer
Source: Plant Direct. 2019 Nov 15;3(11):e00183. doi: 10.1002/pld3.183 (PMC6858605; doi:10.1002/pld3.183)
Supplement: Supplementary file 4 [file PLD3-3-e00183-s004.pdf]

Point-by-point response

Reviewer #1:

I'm responding to several of the author's responses to Reviewer 1, because I feel that the responses were not adequate. Please refer to your point-by-point responses.

1st point, beginning "Figure 3: Measuring the linearity....."

The first part of your reply is indicating that you are going to ignore my suggestion of doing a spike-in experiment. One reason that a spike-in experiment is superior to varying the internal standards is that it mimics the situation of biological samples, in which you are trying to investigate whether you can measure the change in a particular lipid in the case where the ion suppression and the internal standard concentrations remain relatively constant. Regarding the figure, as I mentioned before, the insets should blowup about the lowest 5% of the data. The current insets are meaningless, as they are actually showing the lowest 40-80% of the data SMALLER than in the original figure! It's true that the lowest 5% will not look so linear! That is actually the point of blowing it up - It's so that we can see where the linearity ends at the low end. This will allow you to determine your limit of detection. Below the limit of detection, it won't be linear.

Reply: Thank you for the suggestion. We did the spike-in experiment, analyzed the data and the results and showed the lower concentration results. The results are shown in Fig.3 and Figure S2 and S3.

2nd point, beginning "Lines 264-284: The resolution of the data ....."

I still believe that ppm difference of observed vs theoretical m/z should be calculated, using the formula that I indicated. This is not just a property of the instrument; it indicates the quality of the data. I also believe that you should mention in the text that PA(36:2) {M+NH4]+ and PE(34:1) [M+H]+ have the same chemical formula (C39H77O8PN) and same theoretical m/z. Line 262: Change "could keep a great consistence" to "were consistent" and Line 277: Change "was showed" to "are shown".

Reply: Thanks for the suggestion. We calculated the resolution and added the information in the text. The resolution of the machine for PA-36:2 and PE-34:1 were 2.78 and 4.18 ppm, respectively, and PC-34:1 and PG-34:4 were 0.26 and 2.89 ppm, respectively. We added the information of the PA(36:2) [M+NH4]+ and PE(34:1) [M+H]+ have the same chemical formula and same theoretical m/z in the text, and also corrected the terms.

Point beginning "Have you considered whether the response in comparison to the internal standard is the same for all the lipids in each class?...."

Reply: Lipids in each class have different acyl chains and it is believed that their response could be different for different species. We have not calculated the response using two internal standards for lipid classes. We only used one internal standard for quantification of each lipid class. We agree it would be ideal to use two internal standards with short and long acyl chains to calculate the response factor for each lipid class, which will provide better quantification.

It would be a good idea to discuss the limitations on your quantification.

Reply: Thanks for the suggestion. We have added a few sentences about the limitations (line 402-407).

Point beginning "Method section 2.2: How were these internal standards quantified?......" Please indicate how the internal standards were quantified.

Reply: We added the method in the text. Briefly, internal standards were weighted and quantified as fatty acid methyl esters by GC according to the method described by Welti et al (2002) before preparation of mixed internal standards.

Reviewer #2:

Authors have made sufficient edits to the manuscript to satisfy my concerns and suggestions.
